# Identification and characterization of a novel stay‐green QTL that increases yield in maize

**DOI:** 10.1111/pbi.13139

**Published:** 2019-05-20

**Authors:** Jun Zhang, Kevin A. Fengler, John L. Van Hemert, Rajeev Gupta, Nick Mongar, Jindong Sun, William B. Allen, Yang Wang, Benjamin Weers, Hua Mo, Renee Lafitte, Zhenglin Hou, Angela Bryant, Farag Ibraheem, Jennifer Arp, Kankshita Swaminathan, Stephen P. Moose, Bailin Li, Bo Shen

**Affiliations:** ^1^ Corteva Agriscience, Agriculture Division of DowDuPont Johnston IA USA; ^2^ Department of Crop Sciences University of Illinois Urbana IL USA; ^3^ Botany Department Faculty of Science Mansoura University Egypt; ^4^ Present address: International Crops Research Institute for the Semi‐Arid Tropics Patancheru India; ^5^ Present address: Mansoura University Mansoura Egypt; ^6^ Present address: Donald Danforth Plant Science Center Creve Coeur MO USA; ^7^ Present address: Hudson Alpha Institute for Biotechnology Huntsville AL USA

**Keywords:** yield, QTL, stay‐green, nitrogen remobilization, photosynthesis, proteolysis

## Abstract

Functional stay‐green is a valuable trait that extends the photosynthetic period, increases source capacity and biomass and ultimately translates to higher grain yield. Selection for higher yields has increased stay‐green in modern maize hybrids. Here, we report a novel QTL controlling functional stay‐green that was discovered in a mapping population derived from the Illinois High Protein 1 (IHP1) and Illinois Low Protein 1 (ILP1) lines, which show very different rates of leaf senescence. This QTL was mapped to a single gene containing a NAC‐domain transcription factor that we named *nac7*. Transgenic maize lines where *nac7* was down‐regulated by RNAi showed delayed senescence and increased both biomass and nitrogen accumulation in vegetative tissues, demonstrating NAC7 functions as a negative regulator of the stay‐green trait. More importantly, crosses between *nac7* RNAi parents and two different elite inbred testers produced hybrids with prolonged stay‐green and increased grain yield by an average 0.29 megagram/hectare (4.6 bushel/acre), in 2 years of multi‐environment field trials. Subsequent RNAseq experiments, one employing *nac7* RNAi leaves and the other using leaf protoplasts overexpressing *Nac7*, revealed an important role for NAC7 in regulating genes in photosynthesis, chlorophyll degradation and protein turnover pathways that each contribute to the functional stay‐green phenotype. We further determined the putative target of NAC7 and provided a logical extension for the role of NAC7 in regulating resource allocation from vegetative source to reproductive sink tissues. Collectively, our findings make a compelling case for NAC7 as a target for improving functional stay‐green and yields in maize and other crops.

## Introduction

The demand for yield improvement with new technologies is rising because of increasing global demand for food and feed, and the dwindling supply of arable land available for agriculture (Pingali, 2012). Crops such as corn, soybean, wheat, rice and canola account for over half of total human caloric intake either through direct consumption or the meat products raised on processed seeds. Therefore, a primary focus for recent crop genetic improvement is to produce high‐yielding varieties for future sustainable growth.

Among several strategies contributing to increases in crop biomass and yield, extending the duration of photosynthesis is one of the most effective ways (Richards, 2000). The extended foliar greenness or delayed senescence to maintain more photosynthetically active leaves is referred to as ‘stay‐green’ (Thomas and Ougham, 2014). There are two types of stay‐green: cosmetic and functional. In cosmetic stay‐green, an accumulation of pigments on the surface of the organ is caused by defects in the chlorophyll degradation pathways. In contrast, functional stay‐green is defined as retaining both greenness and photosynthetic competence significantly longer along senescence (Hortensteiner, 2009). Thus, the functional stay‐green trait during the final stage of leaf development is important to increase source strength in grain production.

Plants assimilate carbohydrates and nitrogen in vegetative organs (source) and remobilize them to newly developing tissues during development, or to reproductive organs (sink). Increasing source strength in cereal crops leads to higher grain yield (Gregersen *et al*., 2013). The functional stay‐green trait has been shown to be associated with the transition from the carbon (C) capture to the nitrogen (N) remobilization phase of foliar development (Lee and Tollenaar, 2007). In functional stay‐green plants, the C‐N transition point is delayed, or the transition occurs on time but subsequent yellowing and N remobilization occur slowly. In maize, the stay‐green phenotype has been associated with increases in grain yield. A long‐term breeding programme from the 1930s to 2000s demonstrated that both yield and the duration of greenness of modern maize varieties have been steadily increasing (Duvick *et al*., 2010).

There are highly complex and dynamic regulatory networks controlling senescence induction and progression. In addition to hormone and nutrient‐sensing pathways, a number of transcription factors play important roles in the stay‐green phenotypes (Khan *et al*., 2014; Schippers, 2015). NAC [No apical meristem (NAM), *Arabidopsis* transcription activation factor (ATAF) and cup‐shaped cotyledon (CUC)] family members are well studied senescence‐associated transcription factors (Christiansen and Gregersen, 2014; Kim *et al*., 2016), which are involved in various functions, including embryonic, floral and vegetative development, lateral root formation as well as resistance to a variety of biotic and abiotic stresses (Puranik *et al*., 2012). In major crops, overexpression or silencing of NAC family members revealed their roles in drought resistance (OsSNAC1) and yield increase (OsNAC5) in rice, root development in cotton (OsSNAC1) and grain protein improvement in wheat (TtNAM‐B1 RNAi & TaNAC‐S) (Fang *et al*., 2014; Hu *et al*., 2006; Jeong *et al*., 2013; Liu *et al*., 2014; Uauy *et al*., 2006; Zhao *et al*., 2015). Knocking down SlNAP2 or SlORE1S02 (an ortholog of AtORE1 in tomato) results in more fruits with increased sugar content (Lira *et al*., 2017; Ma *et al*., 2018). In the maize genome, bioinformatics analysis identifies more than 100 nonredundant maize NAC genes unevenly distributed over 10 chromosomes (Lu *et al*., 2015; Peng *et al*., 2015), some of which are closely related to known regulators of plant stress responses.

The Illinois High Protein (IHP) and Illinois Low Protein (ILP) populations were derived from a long‐term selection for grain protein concentration (Moose *et al*., 2004). IHP1 and ILP1 plants also differ greatly for their rate of leaf senescence, with ILP1 leaves exhibiting stay‐green and reduced N remobilization compared to IHP1 (Below *et al*., 2004). In the present study, we identified a QTL associated with leaf senescence and nitrogen remobilization from IHP1 × ILP1 crosses. This QTL was mapped to a single gene that we named *nac7*. We suppressed *nac7* expression by RNAi in maize and proved that *nac7* RNAi delayed leaf senescence and increased grain yield of maize hybrids grown in field trials for 2 years at multiple locations. Our physiological measurements further demonstrated that down‐regulation of *nac7* increased plant leaf area, biomass and nitrogen content. Transcriptome profiling of leaves with altered *nac7* expression indicated that NAC7 regulates genes involved in protein turnover, photosynthesis and trehalose‐6‐phosphate pathways to delay senescence and increase yield in maize.

## Results

### Identification and cloning of a leaf senescence and nitrogen remobilization QTL

**Figure 1:**
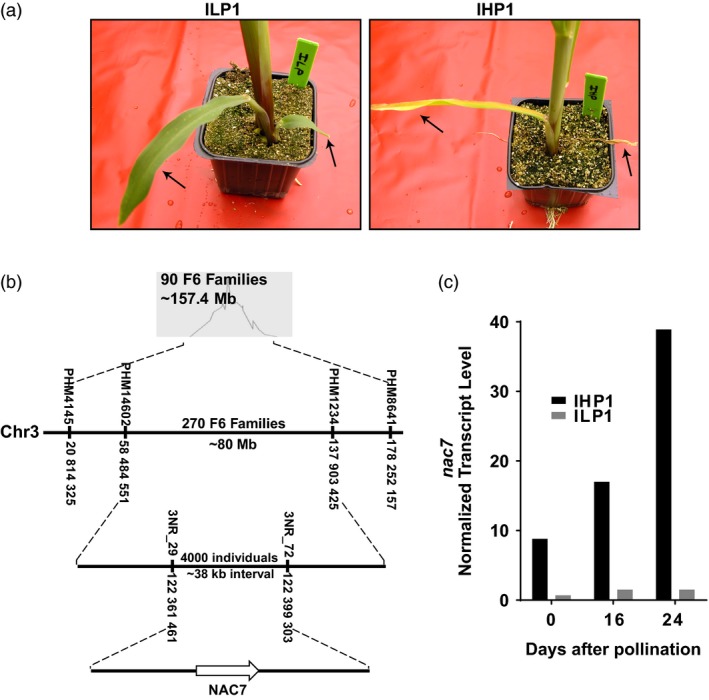
Identification of *nac7,* the candidate gene behind a major stay‐green and nitrogen remobilization QTL. (a) In the nitrogen remobilization seedling assay, Illinois Low Protein 1 (ILP1) delayed leaf senescence when compared to Illinois High Protein 1 (IHP1). (b) Genetic mapping of the F_6_ families showed a strong QTL between SNP markers 3NR_29 and 3NR_72 for stay‐green and nitrogen remobilization. Fine‐mapping delimited the QTL to a 37.84 kb region containing a NAC‐domain transcription factor that we named *nac7*. The locations of SNP markers were based on maize B73 genome assembly V4.0. (c) Transcript of *nac7* in IHP1 and ILP1 was measured by RNAseq at 0, 16 and 24 days after pollination when the plants were grown to maturity.

A clear contrast in the rate of leaf senescence was observed between the IHP1 and ILP1 inbred lines in 4‐week‐old seedlings at the V4 stage (Figure 1a), and these differences were also apparent throughout plant development (Figure S1). A major QTL on chromosome 3 between PHM4145 (B73v4 chr3_20,814,325) and PHM8641 (B73v4 chr3_178,252,157) was detected in F_6_ families derived from the cross of ILP1 with IHP1 (Figure 1b). Two hundred and seventy more F_6_ families from the same population were phenotyped and genotyped to confirm and further refine the QTL interval. The QTL was validated and delimited to the interval between PHM14602 (B73v4 chr3_58,484,551) and PHM1234 (chr3_137,903,425).

To fine‐map and clone the leaf senescence QTL, three F_6_ plants, heterozygous across the QTL interval, were selected for self‐pollination to generate a large mapping population. Five hundred and ninety individuals were initially genotyped with PHM14602 and PHM1234, and 141 recombinants were identified. Subsequently, 3397 individual plants were genotyped with PHM3324 (chr3_100,237,675) and PHM9619 (chr3_126,583,571), and 628 recombinants were identified. The recombinant plants were self‐pollinated, and their progenies were scored for the leaf senescence phenotype. Additional SNP markers were developed within the QTL interval to further genotype the recombinants. The leaf senescence QTL was eventually delimited to a 37.84 kb interval, flanked by 3NR_29 (chr3_122,361,461, 2 recombinants) and 3NR_72 (chr3_122,399,303, 9 recombinants). The recombinants harbouring the ILP1 allele for this QTL co‐segregated perfectly with the delayed leaf senescence phenotype. A single annotated gene encoding a NAC‐domain containing transcription factor was within this interval (Figure 1b). This NAC‐domain containing gene (gene model GRMZM2G114850 or Zm00001d041472, *nactf108* in MaizeGDB) was named as *nac7* based on its subsequent sequence in our testing pipeline. We examined transcript differences of *nac7* between leaves of IHP1 and ILP1 field‐grown plants. Figure 1c shows significantly higher mRNA level of *nac7* in IHP1 compared with ILP1 when sampled at 0, 16 and 24 days after pollination. RNAseq coverage plots illustrate only exon 1 shows mapped reads from *nac7* transcript in ILP1 (Figure S2, upper panel). Sanger sequencing of genomic DNA further demonstrates that *nac7* allele in ILP1 harbours mutations near both splice junctions of exon 2, which could influence RNA splicing and lead truncated *nac7* mRNA in ILP1 (Figure S2, lower panel). Based on the above information, we hypothesize that diminished expression of *nac7* in ILP1 leads to the stay‐green phenotype of the mature leaves.

### Molecular characterization of *nac7*

In the maize genome, there are more than 100 predicted NAC genes (Peng *et al*., 2015). To understand the evolutionary relationship and functional relevance of *nac7* with its homologs, 113 maize NAC protein sequences and 25 well‐known senescence and stress‐related NAC members from *Arabidopsis,* rice, wheat and tomato (Table S1) were used to generate a phylogenetic tree. Figure S2 shows *nac7* is clustered into a broad NAC1 clade that is distant from the NAM or NAP branches. The closest ortholog of *nac7* is OsNAC60, a target of miR164 and negative regulator for drought tolerance in rice (Fang *et al*., 2014).

Comparison of predicted secondary structural elements of NAC7 with ANAC019, whose crystal structure is available (Welner *et al*., 2012), and with five NAC members from other major crops reveals that most of the conserved sequence motifs in the N‐terminal portion of NAC7 are involved in structural integrity and DNA recognition (Figure S4). In its disordered C‐terminal transcription regulatory domain, NAC7 has several molecular recognition features (MoRFs) that may mediate interactions with other transcriptional regulatory proteins (Jensen and Skriver, 2014). A distinguishing feature from other homologs, as shown in Figure S4, is the presence of a PEST motif (RETAPATPPPPLPP), which suggests that NAC7 may be subjected to complex post‐translational regulation.

### RNAi knockdown of *nac7* delays leaf senescence

**Figure 2:**
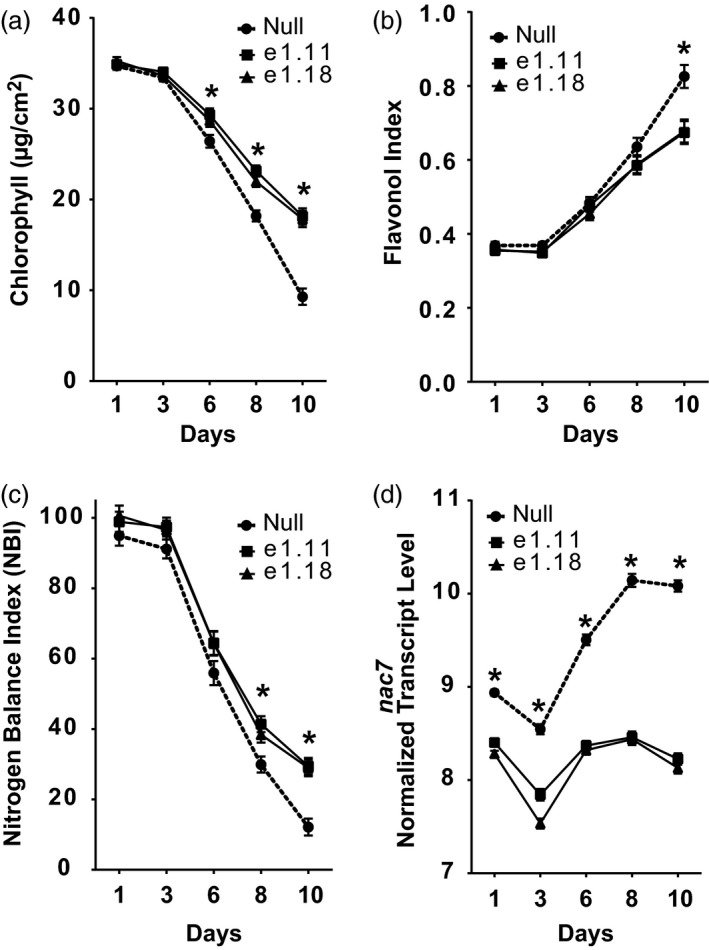
Knocking down of *nac7*, by RNAi, delayed senescence in maize. PHR03 maize line was transformed with a maize ubiquitin (*Ubi*) promoter‐driven *nac7* RNAi construct. (a–c) chlorophyll content, flavonol and nitrogen balance index (NBI) of V3 leaves were measured for 10 days after plants reached V3 stage. Two RNAi events showed higher chlorophyll content and NBI compared with null during the V3 senescence progress under the nitrogen‐free Hoagland's solution. Data are presented as mean ± SD (*n* = 5 plants per time point). (d) Transcript of endogenous *nac7* in V3 stage was measured by RNAseq from day 1 to day 10 after V3 leaves were fully expanded (*n* = 4). Significant difference was determined using the *t*‐test, *P <* *0.05.

A transgenic RNAi approach was employed to test the function of NAC7 in the elite maize inbred line PHR03. Events containing a single‐copy transgene with detectable mRNA expression were selected for phenotyping experiments. RNAseq analysis confirmed the *nac7* RNAi construct significantly knocked down the transcript of native *nac7* in seedling leaves from both transgenic events e1.11 and e1.18 (Figure 2d).

Nitrogen deficiency commonly induces leaf senescence (Schulte auf'm Erley *et al*., 2007). We investigated the impact on leaf senescence from silencing *nac7* in the PHR03 elite line by growing seedlings in hydroponics to the V3 growth stage, challenging them with a low nitrogen treatment and then monitoring physiological indicators of leaf senescence.

During leaf senescence, chlorophyll typically decreases and flavonol production increases, particularly under stress conditions. Therefore, we measured both chlorophyll and flavonol content of V3 leaves of *nac7* RNAi plants using a Dualex instrument. Figure 2a shows leaf chlorophyll decreased when leaves senesced. Leaf chlorophyll level in the null plants declined at a greater rate than that of *nac7* RNAi plants after 3 days of nitrogen‐starved growth. From day 6, leaf chlorophyll level was significantly lower in null plants. Figure 2b shows that after 6 days, leaf flavonol level increased at a greater rate in null compared with *nac7* RNAi plants. At day 10, leaf flavonol was significantly higher in null plants. Leaf nitrogen balance index, a plant health indicator which is derived from the ratio between chlorophyll and flavonol, decreased during leaf senescence. Figure 2c shows that after the 8 days of growth with N limitation, nitrogen balance index was significantly higher in *nac7* RNAi plants. These results indicate that in the elite inbred line PHR03, chlorophyll and flavonol contents were affected by *nac7* silencing, supporting the hypothesis that *nac7* RNAi was able to recapitulate the stay‐green phenotype of ILP1, both of which express *nac7* at lower levels than either the null or IHP1 genotypes.

### *nac7* RNAi events showed stay‐green phenotype under field conditions

**Figure 3:**
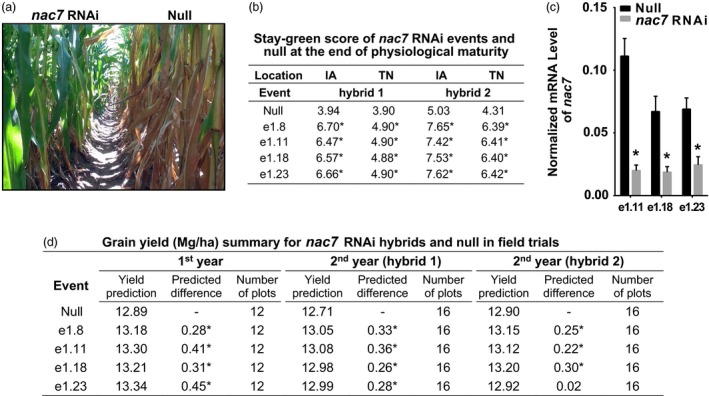
Down‐regulation of *nac7* in maize increased gain yield in two‐year field trials. (a) *nac7* RNAi plants driven by an *Ubi* promoter showed stay‐green phenotype in a yield trial under normal nitrogen condition. Pictures were taken postanthesis. (b) Stay‐green score of two hybrid lines expressing *nac7* RNAi in Iowa and Tennessee with 2 plots per location. Scores ranged from 1 to 9 with ‘9’ being a fully green canopy and ‘1’ being completely senesced with no green. Stay‐green score was analysed by linear unbiased prediction model. Significance between transgenic events and null comparator was determined at the *P *<* *0.1 level shown with *. (c) *nac7* was down‐regulated in three *nac7* RNAi events under field conditions as measured by qPCR (*n* = 8). Transcript level of *nac7* relative to the endogenous reference eIF4g, a maize eukaryotic translation initiation factor, was calculated by the ∆Ct method. Significant difference between each transgenic event and its null was determined using the *t*‐test, *P <* *0.001. (d) Yield tests in two years demonstrated that down‐regulation of *nac7* increased yield in three hybrid lines. Yield was analysed by linear unbiased prediction model and shown as the best linear unbiased predictions (BLUPs). Summary table shows the yield and yield difference between nulls and the transgenic events in megagram/hectare (Mg/ha) at multilocations under normal nitrogen and well‐watered conditions. Statistical significance was determined at the *P *<* *0.05 level shown with *.

After we confirmed *nac7* RNAi delayed seedling leaf senescence in the greenhouse, we investigated whether the transgenic *nac7* RNAi events exhibit a visible stay‐green phenotype in the field under optimal growth conditions. Figure 3a demonstrates the stay‐green phenotype of *nac7* RNAi plants grown under normal nitrogen conditions in the first‐year yield trial. Figure 3b shows the estimated stay‐green scores (1 to 9 score) at physiological maturity for two *nac7* RNAi hybrids grown at two testing locations in Iowa and Tennessee. The down‐regulation of *nac7* to 18%–28% of normal expression levels (Figure 3c) was associated with significant increases in the stay‐green score, which ranged from 4.9 to 7.7 across all events, compared to an average value of 4.3 for the null controls.

### Down‐regulation of *nac7* in maize increased yield

Previous studies have indicated that the functional stay‐green phenotype is associated with increases in crop yield (Thomas and Ougham, 2014). To test whether the delayed leaf senescence conditioned by down‐regulation of *nac7* may improve source capacity and subsequent grain yield, we conducted yield trials for *nac7* RNAi events in 2 years at 10 locations with two replicates per location. Each of three transgenic events containing the construct PHP52729 showed significant increases in yield with 0.28–0.45 Mg/ha (4.5–7.2 bu/ac) across the six locations in the first year of testing (Figure 3d). In the second year, we retested PHP52729 events at more locations with two different inbred testers. Efficacy was repeated with an average yield boost of 0.25 Mg/ha (4.0 bu/ac).

Taken together, results in Figure 3 provide strong evidence that the *nac7* RNAi construct not only delayed plant senescence under field conditions, but also produced consistent yield increases and steady event performance in our two‐year multilocation trials under normal nitrogen and well‐watered conditions.

### Physiological basis of yield efficacy of *nac7* RNAi events

**Figure 4:**
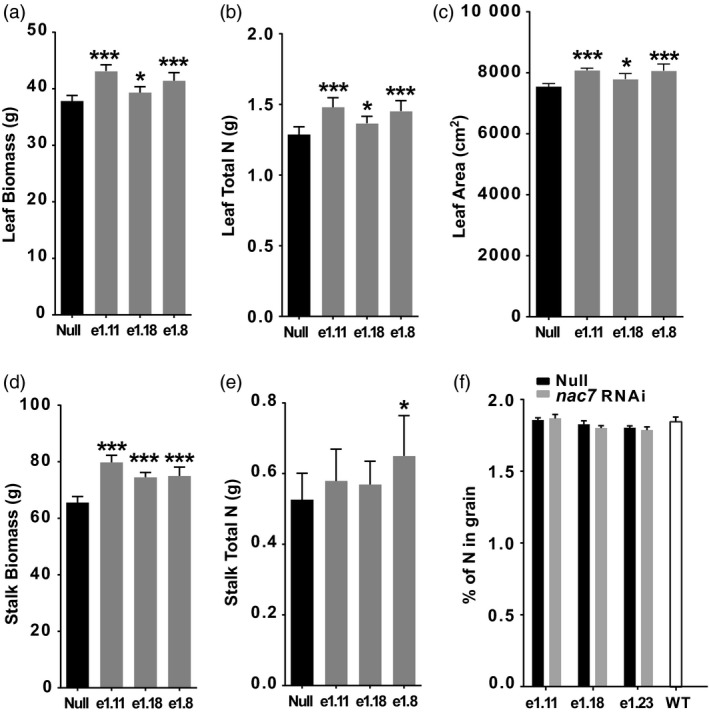
*nac7* RNAi increased biomass and N partitioning in leaf and stalk. Leaf biomass (a), total N in leaf (b), leaf area (c), stock biomass (d) and total N in stalk (e) were measured in PHR03 lines at R1 stage. *n* = 8. Significant difference was determined using the *t*‐test, *P <* *0.05; **P <* *0.01 and ***P <* *0.001. (f) Knocking down *nac7* did not reduce grain nitrogen content in PHR03 line. Grain nitrogen content of 3 RNAi events and nulls was determined by combustion analysis. Event null and 3 RNAi events did not show statistical difference in grain nitrogen compared with that of WT (*n* = 5).

To characterize further how delayed senescence from *nac7* RNAi improves grain yield, we measured biomass and nitrogen partitioning at flowering (R1 growth stage) among leaves, stalks and grain from *nac7* RNAi plants grown under field conditions. We observed that plant leaf biomass at R1 was 9.2% higher on average for the three *nac7* RNAi lines compared with the null plants (Figure 4a), and leaf area was also increased by 5.8% (Figure 4c). An even larger increase of 16.6% was found for stalk biomass (Figure 4d). Additionally, we found that each of the three events with reducing *nac7* mRNA level increased leaf nitrogen content, by 11.3% on average (Figure 4b). Further, there was a trend for increased total nitrogen level in the stalk of *nac7* RNAi lines, although only event 1.8 showed a statistically significant increase (Figure 4e). These findings suggest that reducing *nac7* expression increased plant leaf area, biomass and nitrogen content of the leaf and stalk tissues.

To assess if *nac7* expression level is associated with the grain protein content as it was shown in the ILP1 (Uribelarrea *et al*., 2004), we measured grain nitrogen level in PHR03 lines overexpressing the *nac7* RNAi construct. Interestingly, we did not observe significant changes in grain nitrogen concentration for the *nac7* RNAi lines as measured by combustion analysis. The event null and three RNAi events did not show a statistically significant difference in grain nitrogen content when compared to the WT control, which was 1.84% (Figure 4f).

### Regulation of senescence and photosynthesis‐related genes by *nac7* RNAi

To better understand the molecular mechanisms by which NAC7 regulates the stay‐green phenotype, we collected leaf discs every other day and performed RNAseq analysis from the same experiment shown in Figure 2 that compared V3‐stage seedlings for two *nac7* RNAi events and null controls. A total of 2921 differentially expressed genes (DEGs) were identified from all experimental contrasts, followed by clustering analysis (Figure S5).

The first step in determining if *nac7* RNAi plants were functionally stay‐green was to examine the expression patterns of senescence and photosynthesis‐associated genes. Figure S5a presents transcript changes of three representative genes previously reported as biomarkers for cellular senescence: putative ripening‐related protein 6 (Giovannoni, 2004), autophagy‐related protein 3 (Hanaoka *et al*., 2002) and beta‐galactosidase 13‐like protein (Smith and Gross, 2000). This behaviour was confirmed in our experiment, as the expression of each gene increased as the leaves progress through senescence. However, in *nac7* RNAi leaves, mRNA level of these three genes was significantly lower than nulls after 6 days of the experiment. This result establishes that *nac7* RNAi plants showed a broader delay in the leaf senescence programme, beyond just the visual changes in pigment catabolism.

Photosynthesis‐associated enzymes such as phosphoenolpyruvate carboxylase (PEPC), aspartate transaminase (AST), ribulose bisphosphate carboxylase (RuBisCo) and others in the Calvin cycle (Furbank and Taylor, 1995) play important roles in carbon capture and fixation. From our RNAseq experiment, we found that expression declines over time for PEPC and AST, two enzymes responsible for CO_2_ fixation in mesophyll cells, as well as ribulose‐5‐phosphate epimerase (RPE) that catalyses the epimerization of ribulose 5‐phosphate to xylulose 5‐phosphate in the Calvin cycle. However, at later stages the *nac7* RNAi plants maintain higher expression levels for these genes compared to controls (Figure S6b).

We found that the differences in chlorophyll and flavonoid content previously measured in Figure 2 were reflected in their mRNA expression profiles (Figure S6c). For example, expression of pheophorbide α oxygenase (PAO) that participates in chlorophyll degradation during senescence (Hortensteiner, 2006) was significantly reduced by *nac7* RNAi. Furthermore, expression of flavonoid 3′5′‐hydroxylase (Falcone Ferreyra *et al*., 2012), a key enzyme for flavonol biosynthesis, was decreased too. In addition, expression of the chlorophyll a‐b binding protein was elevated in the very late stage of V3 leaf senescence. Note that, corresponding with the higher chlorophyll level in *nac7* RNAi plants, expression of *nac7* and these senescence‐associated genes were most significantly lower in the late stage of senescence (from day 6 to 10) when compared with nulls.

### *nac7* RNAi events targeted on protein turnover pathways to delay senescence

**Figure 5:**
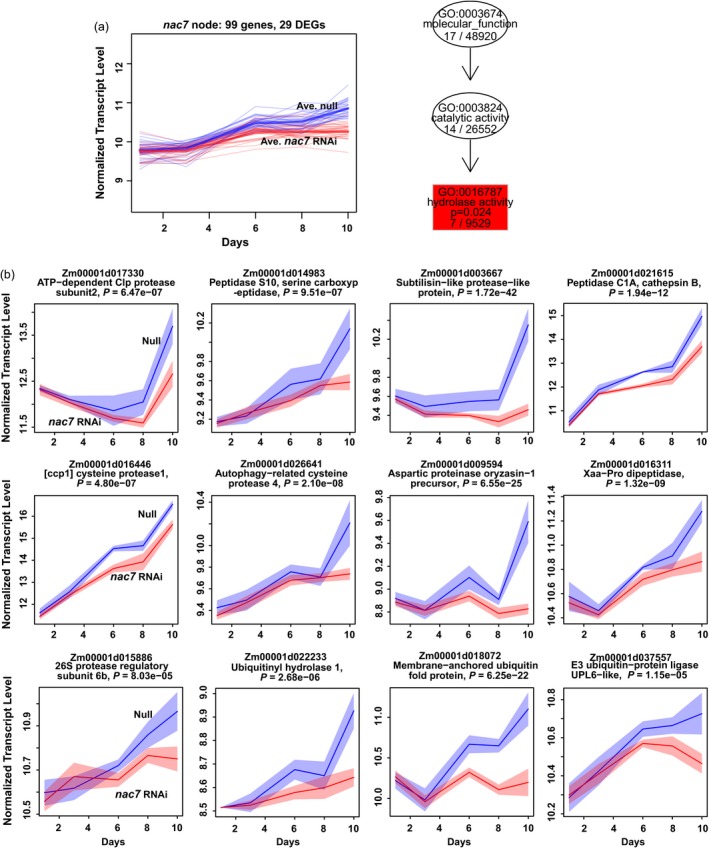
Clustering and enrichment analysis of DEGs showed hydrolase activity (GO:0016787) was down‐regulated by *nac7* RNAi. (a) In the transcriptome of *nac7* RNAi leaf, 99 genes were clustered into the *nac7* node based on their similar expression pattern. Twenty‐nine of them were identified as DEGs. Blue and red thick lines represent average expression pattern of 29 DEGs. Hydrolase activity was shown as the only down‐regulated pathway in *nac7* node with statistical significance (right panel). (b) Representatives of different types of hydrolysis‐related genes in the *nac7* node or its neighbouring nodes were down‐regulated after knocking down *nac7* in 10 days of V3 leaf senescence. Expression profiles for these genes were plotted in the variance‐stabilized log2 count scale, with 95% confidence intervals shaded. Red indicates transgenic events, and blue indicates null controls. ‘*P*=‘ shows the adjusted *P*‐value for the likelihood ratio test (LRT). *n* = 4. MaizeGDB gene model numbers (V4) were listed for each DEGs.

In addition to the above genes whose expression changes were predicted to be associated with the stay‐green phenotype, we further explored how NAC7 regulates senescence by clustering DEGs and performing functional enrichment analysis. As illustrated in Figure S5, each DEG and non‐DEG was assigned to a node on a self‐organizing map (SOM) based on the similarity of their expression patterns. The *nac7* gene was assigned to the node 85. Figure 5a shows expression patterns for 29 DEGs among a total of 99 genes in node 85 containing *nac7*. These 29 DEGs (listed in Table S2) were tested for enrichment of GO terms against non‐DEGs in the same node, which has proven to be an effective test for functional enrichment in DEGs versus other genes (Wehrens and Buydens, 2007), leading to an understanding of a more specific transgene effect in *nac7* RNAi plants because DEGs with inconsistent expression trend in time course of V3 leaf senescence progress were removed. Interestingly, hydrolase activity (GO:0016787) was the only enriched gene ontology (right panel of Figure 5a), which is consistent with prior work documenting that proteolysis and hydrolysis play critical roles during senescence to degrade protein and remobilize nutrients (Roberts *et al*., 2012).

Hydrolase activity refers to enzymes that catalyse the hydrolysis of various bonds and contains 24 direct child terms such as serine hydrolase activity, ubiquitinyl hydrolase activity, peptidase activity, C‐O bond, C‐N bond and C‐C bond hydrolase activity. In the *nac7* node and its neighbours, we found expression of a large group of DEGs involved in proteolysis was significantly lower in *nac7* RNAi plants. Figure 5b displays expression patterns for individual genes from different classes of genes associated with protein turnover as the V3 leaf progresses through senescence. It is worth noting that the ATP‐dependent Clp protease subunit 2 and subtilisin‐like protease, which have been previously associated with later stages of leaf senescence (Roberts *et al*., 2012), were most strongly expressed at days 8 and 10 in control plants, when the greatest reductions were also observed for the *nac7* RNAi plants. In contrast, *nac7*‐dependent expression reduction of aspartic protease, serine carboxypeptidase S10 and ubiquitin‐proteasome components started earlier. Collectively, comparisons of the *nac7* RNAi and control transcriptomes show that NAC7 regulates genes associated with many of the key processes that occur during maize leaf senescence.

### Identification of the putative target of NAC7 in protoplasts

**Figure 6:**
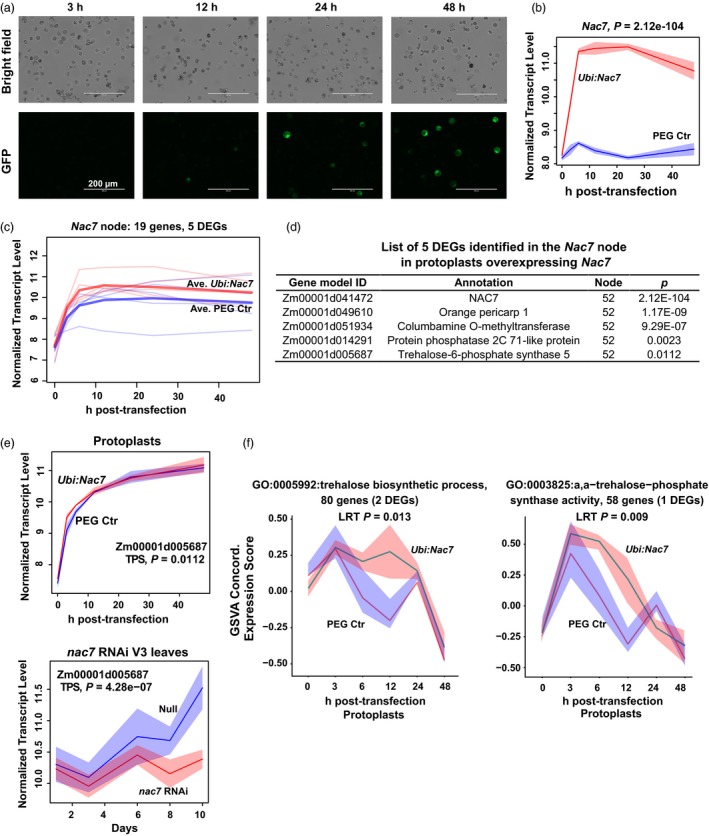
Identification trehalose‐6‐phosphate synthase (TPS) as a putative target of NAC7 in protoplasts. (a) Protoplasts isolated from PHR03 leaf mesophyll cell were transfected with *Ubi:Nac7* and *35S:Ac‐Gfp*. Expression of GFP was monitored in 0‐ to 48‐h post‐transfection. (b) Expression of *Nac7* peaked during the window between 6 and 12 h post‐transfection. *n* = 4 transfections. (c) Transcriptome of transfected cells was compared with that of PEG only control. Nineteen genes were clustered into the *Nac7* node and 5 of them were identified as DEGs. Blue and red thick lines show average expression of 5 DEGs for two treatments. (d) List of 5 DEGs identified in the *Nac7* node. *P*‐value shows statistical significance of the normalized expression difference between protoplasts transfected with PEG only control and *Ubi:Nac7*. (e) The trehalose‐6‐phosphate synthase (TPS) gene showed opposite expression patterns in two RNAseq experiments where NAC7 was either down‐regulated in *nac7* RNAi leaves or overexpressed in protoplasts. (f) Gene Set Variation Analysis (GSVA) determined that trehalose biosynthetic process and α‐α‐trehalose‐phosphate synthase activity categories were positively correlated with overexpressed *Nac7*. Red indicates protoplast overexpressing *Nac7* and blue indicates controls (*n* = 4). 95% confidence intervals are shaded, with adjusted *P*‐value for the likelihood ratio test shown on the top.

The above findings point to the hypothesis that *nac7* RNAi down‐regulated protein turnover pathways and delayed senescence. To further determine how *nac7* RNAi contributed to the observed yield increases (Figure 3d), we utilized mesophyll protoplasts to identify putative targets of NAC7 by RNAseq analysis. A homogeneous protoplast population and transient expression system offered unique advantages to capture early response targets of NAC7. Instead of using the same *nac7* RNAi construct, we transfected protoplasts with a vector where *Nac7* overexpression was driven by the maize *Ubiquitin1* (*Ubi1*) promoter. By comparing the two sets of RNAseq results (*Nac7* overexpression Vs *nac7* down‐regulation by RNAi), the identified targets can be cross‐checked. Our optimized PEG transfection and protoplast culture protocol provided ~80% transfection efficiency (Figure 6a). As shown in Figure 6b, we confirmed overexpression of *Nac7* by 3 h after transfection, and *Nac7* expression peaked during the window between 6 and 12 h post‐transfection.

The RNAseq data set from leaf protoplasts overexpressing *Nac7* was used for clustering analysis (Figure S5b). A total of 1190 DEGs were identified and distributed into 100 nodes. Surprisingly, the node that contains *Nac7* had only 19 genes, and 5 of these were identified as DEGs (including *Nac7*). Figure 6c presents the expression patterns of the 5 DEGs from control and *Ubi:Nac7* transfected cells. Compared to the *nac7* RNAi leaves, the lower number of DEGs identified in the protoplasts may reflect a more pure cell population in our protoplast RNAseq experiment. Because of the small number of DEGs in the *Nac7* node (Figure 6d), it was not possible to assess gene ontology enrichment. Among four DEGs, The *orange pericarp1* (*orp1*) gene encodes the tryptophan synthase β subunit with a role in tryptophan biosynthesis in maize (Wright *et al*., 1992). Two NAC transcription factors (NAC25 and NAM‐B1) showed lower expression in the orange‐pericarp mutant line of pummelo (*Citrus grandis*) (Guo *et al*., 2015). Columbamine O‐methyltransferase methylates the oxygen atom of the secondary metabolites such as phenylpropanoids, flavonoids and alkaloids (Lam *et al*., 2007). Protein phosphatase 2C (PP2C) is one of the most well characterized direct targets of a NAC transcription factor. In *Arabidopsis*, AtNAP worked together with PP2C (SAG113) to control stomatal movement and water loss during leaf senescence (Zhang and Gan, 2012). Remarkably, we also noted that trehalose‐6‐phosphate synthase (TPS) was up‐regulated by NAC7 in leaf protoplasts. TPS catalyses the production of trehalose‐6‐phosphate (T6P), an important signalling metabolite that regulates plant carbohydrate metabolism and perception of carbohydrate availability (Ponnu *et al*., 2011).

We next compared expression patterns of these 4 potential NAC7 targets in two RNAseq experiments where NAC7 was either down‐regulated in *nac7* RNAi leaves or overexpressed in protoplasts. Only TPS (Zm00001d005687) showed opposite expression pattern (Figure 6e), which indicates that TPS may be the putative target of NAC7. Prior work has shown that T6P acts through SnRK1 and bZIP transcription factors as an important regulatory module of carbon assimilation, plant development and maize grain yield (Nuccio *et al*., 2015; Ponnu *et al*., 2011). Interestingly, we observed that one bZIP family member (Zm00001d004857) was down‐regulated by *Nac7* overexpression, and another (Zm00001d030577) was up‐regulated in *nac7* RNAi plants. Collectively, our results suggest that TPS could be a novel target of NAC7, and the T6P pathway may be regulated by NAC7 in maize.

### Validation of NAC7 regulated pathways in protoplasts

To confirm our hypothesis, Gene Set Variation Analysis (GSVA) (Hanzelmann *et al*., 2013) was used to aggregate gene expression into pathway concordant expression scores for all member genes with similar temporal patterns. Further support for the regulation of T6P by NAC7 comes from the results of GSVA analysis with the gene ontology categories of trehalose biosynthetic process (GO:0005992) and α,α‐trehalose‐phosphate synthase activity (GO:0003825), each of which exhibited concordant differentially higher expression in *Nac7* overexpression cells (Figure 6f).

Because reduced *nac7* mRNA level altered photosynthesis, flavonol synthesis and protein turnover pathways, we also investigated the behaviour of these genes following overexpression of *Nac7*. Figure S6a shows genes in the category for photosynthesis (GO:0015979) and its child category light reaction (GO:0019684) were down‐regulated in protoplasts overexpressing *Nac7*, which corresponds well to the up‐regulation of photosynthesis‐associated enzyme genes, especially for those in carbon fixation steps, in *nac7* RNAi plants (Figure S6b).

NAC7 also up‐regulated autophagy (GO:0006914), specifically during 3‐ to 12‐h post‐transfection. However, proteolysis (GO:0006508), when aggregated, did not show a clear up‐regulation with overexpression of *Nac7*. One possibility is that among the large number of genes (2257) included in the proteolysis category, NAC7 may only directly regulate a small portion (32) of them in protoplasts. Only these DEGs could be functionally associated with NAC7 and plant senescence. Taken together, the RNAseq results from protoplasts overexpressing *Nac7* cross‐validated the DEGs and GO categories identified from *nac7* RNAi plants, which strongly support NAC7 functions as a regulator for stay‐green and nitrogen remobilization.

## Discussion

Functional stay‐green is a valuable trait for improving crop yield. It has been shown that delaying leaf senescence directly extends the photosynthetic period and increases source capacity, both of which benefit grain yield increase (Gregersen *et al*., 2013). In modern maize varieties, yield and stay‐green score is positively correlated (Duvick *et al*., 2010). Thus, novel methods to delay leaf senescence and improve crop productivity in plants may contribute to sustainable approaches designed to meet increasing food demand. In this study, we have identified and characterized NAC7 as a negative regulator of the stay‐green trait in maize. Using a combination of approaches including QTL mapping, target gene identification, transgenic validation, nitrogen partitioning studies and agronomic yield trials, we proved that the down‐regulation of *nac7* increased grain yield in maize. Moreover, we propose a mechanism by which NAC7 regulates proteolysis, photosynthesis and trehalose‐6‐phosphate pathways to delay senescence and increase yield.

The initial step in the determination of *nac7* as a stay‐green QTL was to fine‐map and clone the gene from the IHP1 and ILP1 lines, a mapping population derived from the Illinois Long Term Selection experiment (Figure 1). We demonstrated that NAC7 was of primary importance for the stay‐green phenotype of ILP1. There is extensive research about the role of NAC family members in senescence (Kim *et al*., 2016), but this is the first demonstration of a *nac* gene that functions to increase grain yield in maize, one of the most important cereal crops. Here, we provide six lines of evidence that NAC7 plays an essential role in delaying senescence, increasing source capacity and nitrogen partitioning and ultimately improving grain yield in maize. First, *nac7* RNAi plants recapitulated the stay‐green phenotype that we observed in ILP1 lines. Silencing *nac7* in maize delayed senescence in both seedling leaves of plants grown with limiting nitrogen in a greenhouse environment and in mature plants grown with normal nitrogen supply in the field (Figure 2 and Figure 3a,b). Second, we confirmed the negative correlation between *nac7* expression and chlorophyll content in leaves (Figure 2a) and up‐regulation of key photosynthesis enzymes in *nac7* RNAi leaves (Figure S6b). Third, *nac7* RNAi plants showed an increase in biomass and nitrogen partitioning both in leaves and stems at the R1 stage when compared with nulls (Figure 4). Fourth, two‐year yield trials in 10 locations in North America demonstrated consistent yield increases in hybrid plants expressing the *nac7* RNAi construct (PHP52729). Multiple PHP52729 events crossed to two different inbred testers produced hybrids that showed 0.29 Mg/ha (4.6 bu/ac) yield increases on average, relative to null controls (Figure 3d). This proved the yield efficacy of *nac7* was reproducible in various field environments and genetic backgrounds. Finally, *nac7* RNAi maize showed normal protein content in the grain as measured by combustion analysis (Figure 4f). Although the higher expression of *nac7* in IHP1 leads to a fast senescing phenotype in the older leaves, which could reflect more rapid nitrogen remobilization, our results clearly indicate that knockdown of *nac7* in maize did not alter nitrogen concentration in grain. Because of the yield increase, the total nitrogen in grain per plant is higher in *nac7* RNAi lines when compared to nulls. Taken together, these data prove *nac7* as a uniquely efficacious gene for increasing maize yield by delaying senescence and increasing source capacity.

Although the paradigms for the regulation of stay‐green have been shaped by well‐studied chlorophyll biosynthesis pathways, the underlying complexity of protein degradation and nitrogen remobilization processes from the senescing tissues remains largely unknown. Therefore, in addition to characterizing the phenotypic and physiological consequences after silencing *nac7*, comparative transcriptomic profiling was performed to gain insights into the gene regulatory network associated with *nac7*. Of the 2921 DEGs identified from the transcriptome of the *nac7* RNAi line during its leaf senescence process, 127 were involved in protein degradation, particularly in the categories of autophagy, serine protease, cysteine protease, aspartic protease and ubiquitin‐related protein turnover. Clustering and enrichment analysis showed hydrolase activity was the only differentially regulated GO category with statistical significance in the *nac7* node (Figure 5a). It was reported that several serine proteases were up‐regulated in senescing leaves of barley and rapeseed (Roberts *et al*., 2012). Senescence‐induced aspartic protease CND41 affects chloroplast degradation and nitrogen remobilization (Kato *et al*., 2004). A strong negative correlation between CND41 and RuBisCo expression in *Arabidopsis* leaves during senescence was also observed (Diaz *et al*., 2008). Further, the two most abundantly expressed transcripts in senescing *Arabidopsis* leaves are cysteine proteases (Guo *et al*., 2004). All of this recent genetic and biochemical evidence suggests that the modulation of protease‐associated proteolysis in plants is a tightly controlled process. For example, protease activities can be directly regulated by controlling protease transcription. Three NAC genes in barley were reported to bind to the cysteine protease gene AK358908 at the NAC binding motif in its promoter (Christiansen and Gregersen, 2014). While further rigorous testing is needed, our comparative transcriptomic data suggest that both proteolysis and autophagy processes could be directly regulated by NAC7. For example, the aspartic peptidase (Zm00001d051695), a member of the aspartic protease family that showed opposite expression pattern in two RNAseq experiments, indeed has a conserved NAC7 bindings motif ‘CGTG’ in its promoter region close to the TATA box.

There is increasing evidence that autophagy is not only associated with cell death, but may also be a triggering factor for senescence onset or a process induced by senescence to facilitate nutrient remobilization (Avila‐Ospina *et al*., 2014). In *Arabidopsis* and maize, recent work demonstrated the role of autophagy in nutrient recycling and nitrogen remobilization to sinks tissues (Guiboileau *et al*., 2012; Li *et al*., 2015). In our experiments, the down‐regulation of the autophagy pathway in *nac7* RNAi line was cross‐validated by the GSVA pathway enrichment analysis for protoplasts overexpressing *Nac7* (Figure S7b), which suggests that autophagy also plays a critical role in NAC7‐regulated senescence in maize.

It has long been established that delayed senescence, which prolongs maximal photosynthetic activity, could improve yield (Thomas and Stoddart, 1980). Accumulating knowledge of NAC transcription factors makes them attractive targets for improving maize yield. However, we found that although manipulating expression level of senescence‐associated NAC members in maize can extend the photosynthetic period, it does not always result in yield increases under field conditions. For example, ZmNAP1 and ZmNAC1, two NAC members that belong to separate branches from NAC7 in the phylogenetic tree of maize NAC transcription factors (Figure S3), did not deliver yield efficacy in our transgenic testing pipeline (data not shown), which indicates functional diversity of the NAC members. While the complete molecular mechanism underlying the yield efficacy of *nac7* RNAi remains to be established, our findings demonstrate that, besides directly affecting senescence, the function of NAC7 may be more complex than originally anticipated. As one example, we found that TPS could be a novel target of NAC7. In two RNAseq data sets, we generated to understand the NAC7‐regulated gene network, TPS expression level was highly correlated with both increases and decreases in *nac7* expression (Figure 6e). Perhaps more importantly, our GSVA enrichment analysis demonstrated that both GO categories of TPS activity and trehalose biosynthetic process were significantly up‐regulated by NAC7 in protoplasts (Figure 6f).

TPS catalyses the first step in the trehalose biosynthetic pathway in plants by producing T6P that is subsequently dephosphorylated by T6P phosphatase (TPP) to trehalose. T6P is an important signalling metabolite that has been shown to regulate leaf senescence, carbon assimilation, sugar utilization as well as plant development and reproduction (Ponnu *et al*., 2011). Decreased T6P delays the onset of leaf senescence (Ponnu *et al*., 2011). Furthermore, one of the current models for T6P in regulating plant growth and yield is primarily based on T6P functioning as a nutrient‐sensing factor, which directs sugar from source to sink tissues for plant growth. Overexpressing TPP and decreasing T6P level maintained photosynthesis rate in leaves, increased sugars and amino acids in florets and substantially improved yield in maize (Nuccio *et al*., 2015; Oszvald *et al*., 2018). Although TPP is an important regulator for T6P, here we present evidence that the T6P level also could be controlled by NAC7 through TPS. Moreover, there is a conserved NAC binding motif ‘CGTG’ at 246 bp upstream of the start codon of TPS. While our work does not exclude contribution from other signalling pathways that may operate concurrently, such as those mediated by PP2C that also is a potential target for NAC7, our investigation provides a logical extension for the biological role of NAC family members in source to sink communication, by regulating the T6P pathway. This additional role of NAC7 fits well with the different mechanisms underlying the stay‐green phenotype and its relationship to improve yield, allowing a single transcription factor to perform its roles in the recruitment and reallocation of cellular nitrogen from senescing parts of the plant to developing seeds for yield improvement.

In conclusion, although NAC transcription factors are well studied, there are few comprehensive reports on their regulation of stay‐green and potential for yield improvement in major crops. In the present study, we identified NAC7 as a novel NAC family member by QTL mapping and then proved that down‐regulation of *nac7* delayed senescence and increased yield in maize in multiyear, multilocation yield testing. These results outlined a new hypothesis for NAC7 in regulating stay‐green by delaying proteolysis and hydrolysis, and directing resource translocation from source to sink via the T6P signalling pathway. Similar approaches to down‐regulate the expression of *nac7* orthologs in other C3 or C4 crops by RNAi or CRISPR technology could provide a means to significantly increase their yield.

## Experimental procedures

### Plant materials and growth conditions

The Illinois High Protein 1 (IHP1) and Illinois Low Protein 1 (ILP1) inbred lines (Uribelarrea *et al*., 2004) used for QTL mapping were provided by Dr. Stephen Moose of the University of Illinois. These lines were grown in field plots at the Crop Sciences Research and Education Center in Urbana, Illinois. Plants grown with either no supplemental nitrogen (low N) or nitrogen fertilizer (high N) at a rate of 100 kg/ha were monitored for leaf senescence at multiple growth stages for the number of leaves with visible loss of chlorophyll from more than 50% of total leaf area. From these same plots, the basal 2 cm of the fully expanded leaf blade was collected for RNA expression analysis.

Elite maize PHR03 lines overexpressing *nac7* RNAi construct were created (see below) and grown in a greenhouse in 4‐inch pots filled with Fafard 3B soil mix for leaf senescence measurements and RNAseq sampling. The plants were arranged as a randomized complete block with 12 pots around the perimeter of a 15‐well pot rack leaving the centre three spaces empty to equalize the light received by the plants. Racks were placed in shallow lidless tubs to be subirrigated with modified Hoagland solution (Sun *et al*., 2002), which consisted of 1 mm MgSO_4_, 0.5 mm KH_2_PO_4_, 1 mm CaCl_2_, 84 ppm Sprint Fe330, 3 μm H_3_BO_3_, 2 μm MnCl_2_, 2 μm ZnSO_4_, 1 μm CuSO_4_, 0.12 μm NaMoO_4_ and 4 mm KNO_3_. When 50% of the plants reached V3 stage, irrigation was switched to modified Hoagland solution without KNO_3_ to promote N‐starvation‐induced leaf senescence.

Hybrid maize plants overexpressing *nac7* RNAi were grown in the field with normal agronomic practice to measure plant biomass and nitrogen content at the R1 stage.

### Identification and cloning of a leaf senescence and N remobilization QTL

The IHP1 and ILP1 were crossed, and the progeny were selfed for 6 generations to generate populations for QTL mapping. The leaf senescence phenotype was scored visually (1 = IHP1‐like, fully senescenced and yellow, 3 = ILP1‐like, not senescenced and green) in 90 families from an IHP1 × ILP1 F_6_ population. The F_6_ population was genotyped with 239 polymorphic SNP markers. Quantitative trait locus mapping was conducted with Windows QTL Cartographer V2.5 (Wang *et al*., 2012). To fine‐map and clone the leaf senescence QTL, three F_6_ plants that were heterozygous across the QTL interval were selected for self‐pollination to generate a large mapping population.

### Phylogenetic and functional domain analysis of NAC7

All the maize NAC sequences were downloaded from the Plant Transcription Factor database (Jin *et al*., 2017) and underwent a redundancy reduction at 95% identity threshold with CD‐HIT (Fu *et al*., 2012). The resulting nonredundant sequences plus the NAC domain identified from the crystal structure of the ANAC019 protein (Welner *et al*., 2012) were aligned together with ClustalW. To ensure 3D structural integrity, all the sequences with incomplete NAC domain were purged. A total of 113 unique and complete maize sequences and 25 well‐known NAC proteins from other species were subject to phylogenetic analysis with MEGA 6.06 (Tamura *et al*., 2013). The sequences were aligned with MUSCLE (Edgar, 2004), and a phylogenetic tree was built with the maximum likelihood method.

For secondary structural elements and functional motifs analysis, the amino acid sequence of NAC7 was aligned with ANAC019, OsSNAC1, SlNAP2, SlORE1S02, TtNAM‐B1 and TaNAC‐S (Fang *et al*., 2014; Hu *et al*., 2006; Liu *et al*., 2014; Uauy *et al*., 2006; Zhao *et al*., 2015) that have shown functional relevance to yield increase in major crops. MoRF (molecular recognition features) were predicted by MORFpred, and the PEST motif was identified by Epestfind.

### *nac7* RNAi construct

A transgenic RNAi approach was used to elucidate the function of NAC7 in an elite maize inbred line PHR03. A suppression DNA construct containing a 310 bp fragment of *nac7* (nucleotides 212–522 of the coding sequence), in the sense and antisense orientation, with potato LS intron 2 as a spacer, was prepared. The RNAi cassette with inverted repeats was driven by the *Zm‐Ubi* promoter and was operably linked to the sorghum bicolor gamma kafirin terminator (SB‐GKAF). The plasmid vector PHP52729 containing the suppression DNA construct also had phosphomannose isomerase (PMI) and maize‐optimized phosphinothricin‐N‐acetyltransferase (MOPAT) driven by maize *Ubi* and rice *Actin* promoters as selectable markers, respectively. *Agrobacterium tumefaciens* containing the suppression DNA construct was used to transform maize to examine the resulting phenotype.

### Leaf chlorophyll and flavonol measurement

When the plants reached V3 stage and the 3rd leaf became fully expanded, leaf senescence was measured at day 1, 3, 6, 8 and 10 until the 3rd leaf was fully senesced. At each time point, leaf chlorophyll content, flavonol and nitrogen balance index (NBI, the ratio between chlorophyll and flavonols) of the 3rd leaf were determined using a Dualex 4 Scientific meter (FORCE‐A, Orsay, France).

### Total nitrogen analysis for leaf and grain tissues

Tissue total nitrogen content was determined by combustion analysis on a Flash 1112EA analyzer (Thermo) configured for N/Protein determination as described by the instrument manufacturer. Dried, finely ground tissue powder samples (25–35 mg; weighed and recorded to an accuracy of 0.001 mg on a Mettler‐Toledo MX5 microbalance) were prepared in tin capsules prior to analysis. Per cent N values were determined using the Eager 300 software (Thermo Scientific, Milan, Italy) based on a calibration of known standards.

### Hybrid yield testing of *nac7* RNAi events

Transgenic events of the *nac7* RNAi construct were characterized for transgene copy number and expression by genomic PCR and RT‐PCR, respectively, to select single‐copy events for field yield testing. The null segregants were used as the null controls. Hybrid seeds were produced with one inbred tester in 1st year and two testers in 2nd‐year field trial. Transgenic events and null controls were grown in two‐row plots at research centres in Johnston, Iowa; Marion, Iowa; Dallas Center, Iowa; Macomb, Illinois; Princeton, Illinois; Windfall, Indiana; Plainview, Texas; Union City, Tennessee; York, Nebraska and Woodland, California. Yield data were collected with two replicates per location.

Analyses of grain yield from normal nitrogen and well‐watered conditions were performed jointly across all locations by a linear mixed model with construct, location and interaction between construct and location as fixed effects. The event, the interaction of event with location, the interaction of row with location, the interaction of column with location and block within location were each analysed as random effects. The heterogeneous residual variance and a separable AR1 structure for both row and column directions were fitted for each location. All analyses were implemented using ASReml (Gilmour *et al*., 2008). The mixed model with spatial adjustment reduced the impact of noise caused by field variability while preserving the genetic signal. The yield value is shown as the best linear unbiased predictions (BLUPs) for the difference from the null in megagram/hectare (Mg/ha).

### Stay‐green analysis of *nac7* RNAi events

Visual stay‐green scores were collected in two testing locations in Iowa and Tennessee, with 2 plots per location. Scores ranged from 1 to 9 with ‘9’ being a fully green canopy and ‘1’ being completely senesced with no green. The scores were taken near the end of physiological maturity where optimal differences in canopy senescence can be observed. Two hybrids, hybrid 1 and hybrid 2, were used to assess potential transgene by genetic background interaction. Data were analysed by ASREML and BLUPs were calculated for all entries.

### Protoplast isolation and transfection

Maize PHR03 seedlings were grown in Fafard Super Fine Germination Mix for 5 days in a growth chamber (30 °C, 60% RH, 24 h light) before being transferred to a dark growth chamber (30 °C, 60% RH, 0 h light) for additional 5 days to obtain etiolated seedlings. These seedlings were subirrigated with deionized water as needed. Protoplasts were isolated and transfected with 10 pmols of *Ubi:Nac7*,* 35S:Ac‐Gfp* or a blank control per 3.0 × 10^4^ protoplasts (Yoo *et al*., 2007). Four plasmids by time point replicates were generated and incubated on 12‐well plates. Transfection efficiency was examined by the GFP marker and qPCR for exogenous *Nac7* expression. Cells were harvested 3, 6, 12, 24 and 48 h after transfection and were flash frozen in liquid nitrogen before stored at −80 °C. Protoplast RNA was isolated using a RNeasy Mini kit (Qiagen) for RNAseq analysis.

### RNAseq for maize leaf and protoplasts

After the plants reached V3, six leaf punches were taken from the 3rd leaf of each plant. These were immediately frozen in liquid nitrogen and stored at −80 °C until processed for RNAseq analysis. Total RNA was isolated from frozen tissues using a Qiagen RNeasy Kit for total RNA extraction. Libraries from total RNA were then prepared using the TruSeq mRNASeq Kit (Illumina) and sequenced on an Illumina HiSeq 2500 system.

### Clustering analysis of RNAseq data

RNASeq data were aligned to proprietary Maize B73 reference gene models using Bowtie 2 (Welner *et al*., 2012), and transcript abundances were quantified with RSEM (Li and Dewey, 2011). Expression was modelled for hypothesis testing using a likelihood ratio test (LRT) in DESeq2 (Love *et al*., 2014) with the following full (accounts for transgene) and reduced (ignores transgene) model designs:

Full:expr=time+transgene+time×transgene

Reduced:expr=time

Proprietary gene model identifiers were annotated with putative Gene Ontology (GO) terms (Ashburner *et al*., 2000) and converted to a public gene model (maize B73 genome assembly V4.0 (Andorf *et al*., 2016) using reciprocal BLAST hits.

Since the likelihood ratio test discovers many different expression patterns, clustering helped organize genes into groups. Self‐organizing maps (SOMs) were used to cluster genes such that *nac7* expression and functional categories could be compared to groups of genes at different levels of granularity. A SOM was employed for each data set (*nac7* RNAi and overexpression) using the following steps: treatment counts were normalized using DESeq2's variance stabilizing transformation. Next, differentially expressed genes (DEGs) were used to train a SOM using a sum of squares distance measure (equivalent to a squared Euclidean distance) in the R (RCoreTeam, 2014) package kohonen (Wehrens and Buydens, 2007). Then, each expression profile in the transcriptome, including non‐DEGs, was assigned to its best‐fitting SOM node, and the DEGs in each cluster were tested for GO enrichment against the rest of the genes in the cluster using the topGO (Wehrens and Buydens, 2007) R package. Thus, functional enrichment was conditioned on behaviour, that is DEGs versus other genes which behave similarly, but lack a specific transgene effect.

### Pathway enrichment analysis

Additionally, Gene Set Variation Analysis (GSVA) (Hanzelmann *et al*., 2013) was used to aggregate individual expression profiles into gene set concordant expression scores. The GSVA Concordant Expression Scores represent a profile for the set. Gene sets analysed this way include genes annotated with specific GO terms found to be enriched with SOM nodes and clusters near *nac7*. Likelihood ratio tests were performed on GSVA profiles similarly to those of individual genes, except scores were modelled using the R Generalized Linear Model (glm) function. GSVA Concordant Expression Scores and LRT results were plotted for GO categories.

## Conflict of interest

This work was funded by DuPont Pioneer, a for‐profit agricultural technology company, as part of its research and development programme. J.Z., K.F., J.L.V.H., R.G., N.M., J.S., W.A., B.W., R.L., H.M., Z.H., A.B., B.L. and B.S. were DuPont Pioneer employees when contributing to this work.

## Supporting information


**Figure S1** Comparison of the senescence phenotype of ILP1 and IHP1 lines across their growth stages.
**Figure S2** *nac7* sequence variation between ILP1 and IHP1 line.
**Figure S3** Phylogenetic tree of putative NAC transcription factors from maize together with the senescence or stress‐related NAC family members from *Arabidopsis thaliana, Oryza sativa, Solanum lycopersicum and Triticum*.
**Figure S4** Functional domains of NAC7, ANAC019 and selected NAC family members from rice (OsSNAC1), wheat (TtNAM‐B1 and TaNAC‐S) and tomato (SlORE1S02 and SlNAP2).
**Figure S5** A self‐organized map (SOM) shows clustering of DEGs regulated by NAC7 in two RNAseq experiments: *nac7* RNAi leaves (a) and protoplasts overexpressing *Nac7* (b).
**Figure S6** *nac7* RNAi increased expression of photosynthesis‐associated enzyme genes when compared to the null.
**Figure S7** Gene Set Variation Analysis (GSVA) showed regulation of photosynthesis, autophagy and proteolysis pathways by NAC7 in protoplasts.

**Table S1** Sequence data from this article can be accessed under the following accession numbers.
**Table S2** List of DEGs in the *nac7* node identified from *nac7* RNAi leaves by RNAseq.
