# Supplementary material for: Identification and characterization of a novel stay‐green QTL that increases yield in maize
Source: Plant Biotechnol J. 2019 May 20;17(12):2272–85. doi: 10.1111/pbi.13139 (PMC6835130; doi:10.1111/pbi.13139)
Supplement: Supplementary file 1 — Figure S1 Comparison of the senescence phenotype of ILP1 and IHP1 lines across their growth stages. Figure S2 nac7 sequence variation between ILP1 and IHP1 line. Figure S3 Phylogenetic tree of putative NAC transcription factors from maize together with the senescence or stress‐related NAC family members from Arabidopsis thaliana, Oryza sativa, Solanum lycopersicum and Triticum. Figure S4 Functional domains of NAC7, ANAC019 and selected NAC family members from rice (OsSNAC1), wheat (TtNAM‐B1 and TaNAC‐S) and tomato (SlORE1S02 and SlNAP2). Figure S5 A self‐organized map (SOM) shows clustering of DEGs regulated by NAC7 in two RNAseq experiments: nac7 RNAi leaves (a) and protoplasts overexpressing Nac7 (b). Figure S6 nac7 RNAi increased expression of photosynthesis‐associated enzyme genes when compared to the null. Figure S7 Gene Set Variation Analysis (GSVA) showed regulation of photosynthesis, autophagy and proteolysis pathways by NAC7 in protoplasts. [file PBI-17-2272-s001.pdf]

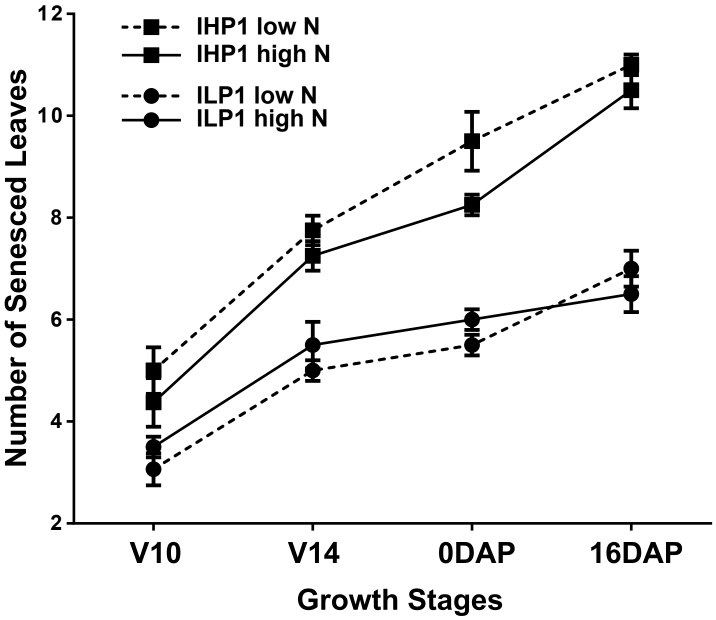

Supporting Figure 1 Comparison of the senescence phenotype of ILP1 and IHP1 lines across their growth stages. Seeds were planted in the field under low N and high N. The number of senesced leaves on each plant was recorded at V10, V14, anthesis (0DAP), and 16 day after pollination (DAP). Data are presented as mean ± s.d. (n=4 plants per time point).

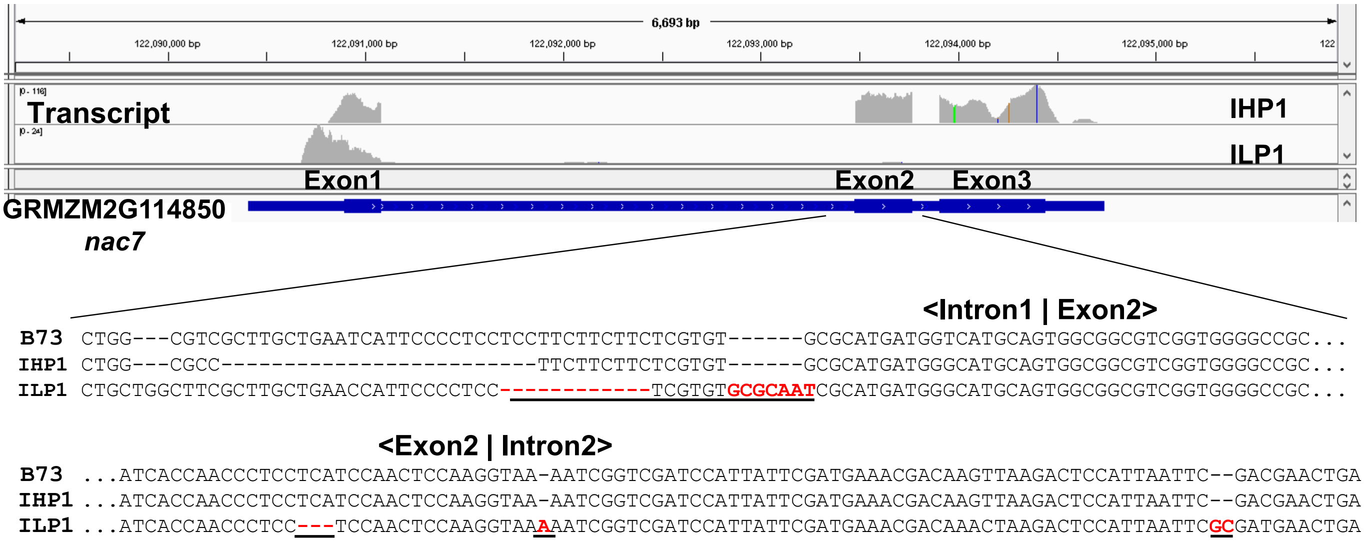

Supporting Figure 2 *nac7* sequence variation between ILP1 and IHP1 line. Top panel displays RNASeq coverage of the *nac7* transcript from 24 DAP leaves of IHP1 and ILP1, following alignment to the B73v3 genome assembly. Lower panel shows the genomic DNA sequences for the region surrounding the splice junctions of exon 2, where variants unique to ILP1 are highlighted in red and underlined. ILP1 harbors mutations near both splice junctions that lead to truncated *nac7* mRNA.

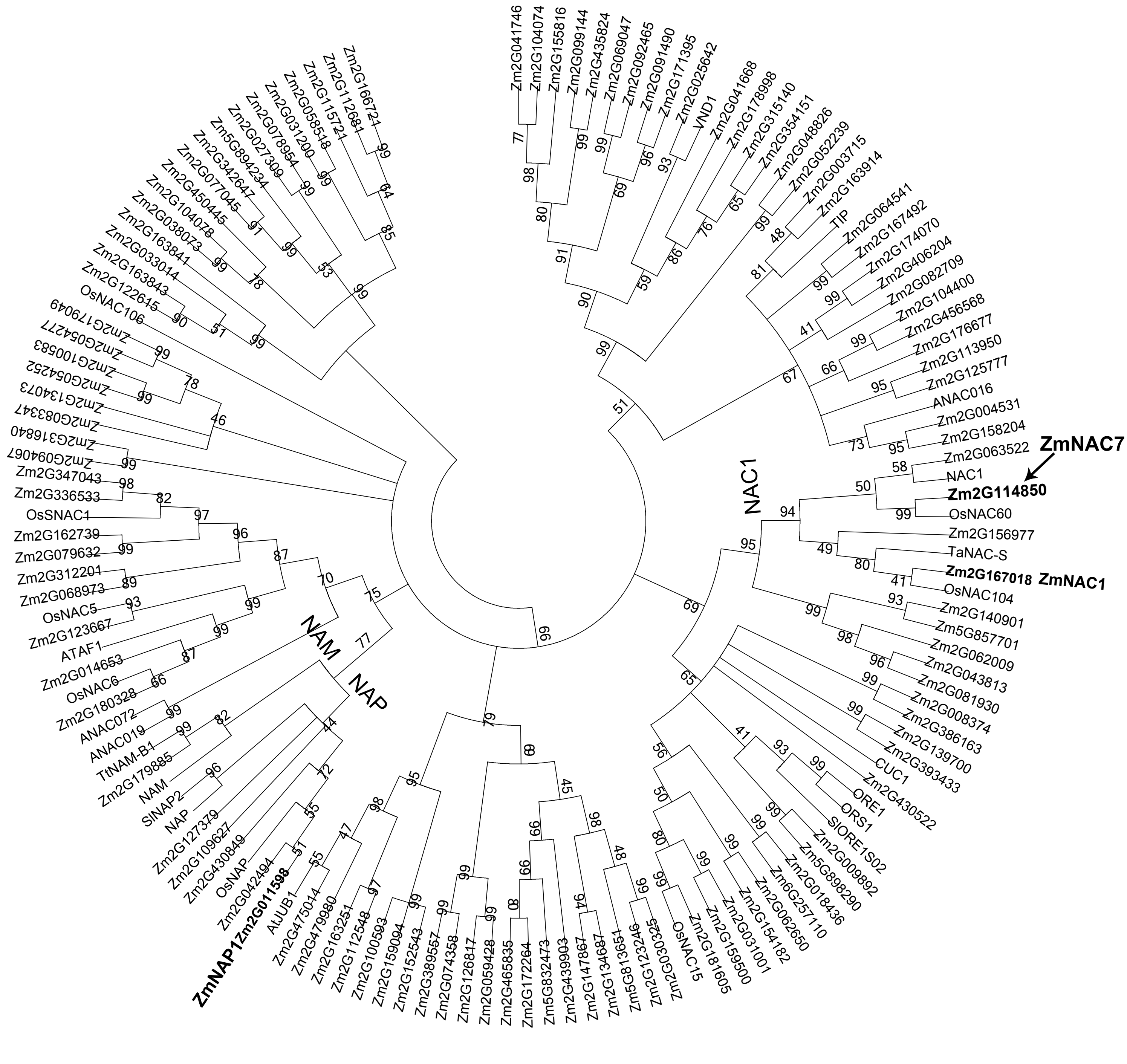

Supporting Figure 3 Phylogenetic tree of putative NAC transcription factors from maize together with the senescence or stress related NAC family members from *Arabidopsis thaliana, Oryza sativa, Solanum lycopersicum and Triticum.* Full length protein sequences were aligned using MUSCLE program with default parameters in MEGA 6 and the phylogenic tree was reconstructed by Maximum Likelihood method with a substitution model of Jones-Taylor-Thornton (JTT). Bootstrap values from 1000 replicates are indicated at each node. NAC7 was clustered into a broad NAC1 clade but distinct from NAM and NAP clade.

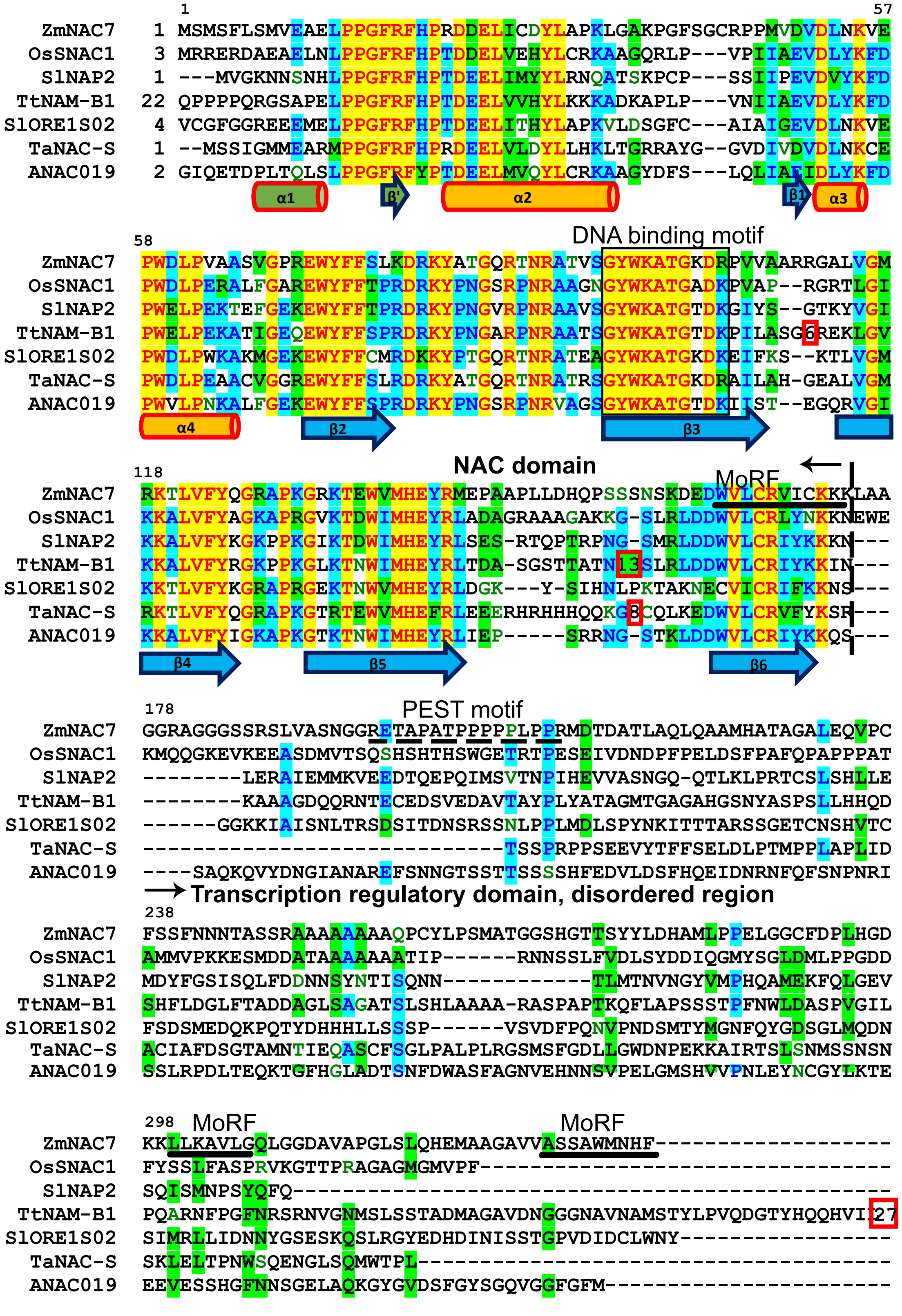

Supporting Figure 4 Functional domains of NAC7, ANAC019 and selected NAC family members from rice (OsSNAC1), wheat (TtNAM-B1 and TaNAC-S) and tomato (SlORE1S02 and SlNAP2). A vertical dash line divides conserved N-terminal NAC domain and disordered transcription regulatory domain. Based on the crystal structure of the ANAC019, a conserved DNA binding motif was determined and shown in a box. Six β-sheets and four α-helices were labeled under the corresponding amino acid sequence. MoRF (molecular recognition features) in the regulatory domain were predicted by MORFpred. A putative PEST motif, potential target site for proteolytic degradation, was identified by Epestfind.

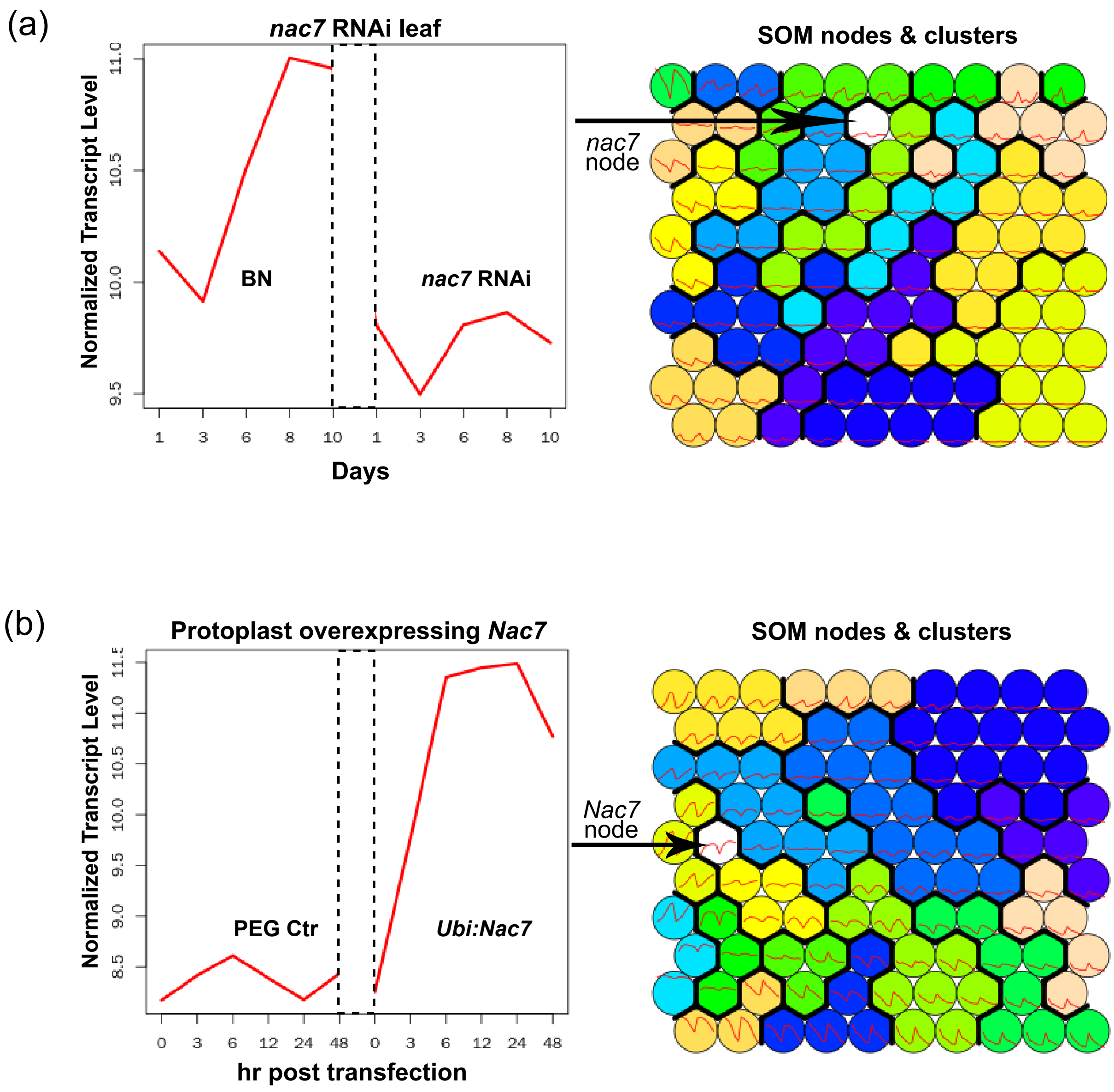

Supporting Figure 5 A self-organized map (SOM) shows clustering of DEGs regulated by NAC7 in two RNA-seq experiments: *nac7* RNAi leaves (a) and protoplasts overexpressing *Nac7* (b). Each DEG was assigned a node on the SOM. Each node is a gene cluster with similar expression pattern. *nac7* is in the node 85 and *Nac7* is in the node 52, which has white background and appears as a circle on a grid. Left panels shows expression pattern of *nac7* and *Nac7* plotted together with their controls, which has been used to align with other transcripts from two RNAseq datasets for clustering analysis.

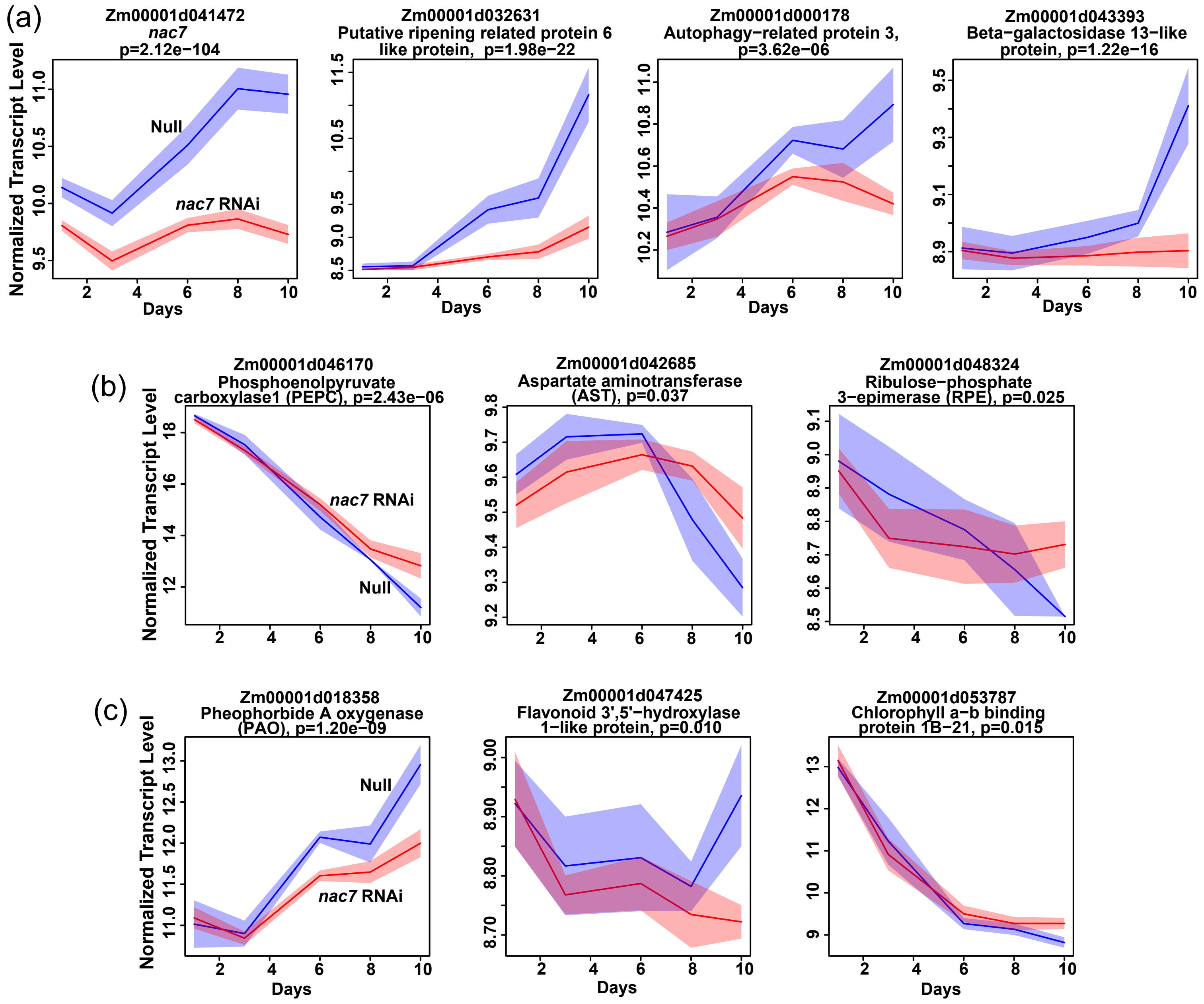

Supporting Figure 6 *nac7* RNAi increased expression of photosynthesis-associated enzyme genes when compared to the null. (a) Transcriptome comparison of the null and two RNAi events showed a negative correlation between expression of three representative senescence associated genes and *nac7* during V3 leaf senescence progress. (b) Phosphoenolpyruvate carboxylase 1 (PEPC), Aspartate aminotransferase (AST) and Ribulose−phosphate 3−epimerase (RPE), three key enzymes involved in carbon capture and fixation in photosynthesis, showed higher expression in *nac7* RNAi plants in 10 days of V3 leaf senescence when compared to these in the null. (c) *nac7* RNAi reduced expression of Pheophorbide A oxygenase (PAO) and Flavonoid 3',5'-hydroxylase that control chlorophyll degradation and flavonol biosynthesis, respectively. Expression of chlorophyll a/b binding protein was higher in the late stage of V3 leaf senescence of *nac7* RNAi plants when compared to that of null. Red indicates transgenic events and blue indicates null controls. "*p*=" shows the adjusted *p*-value for the Likelihood Ratio Test (LRT).

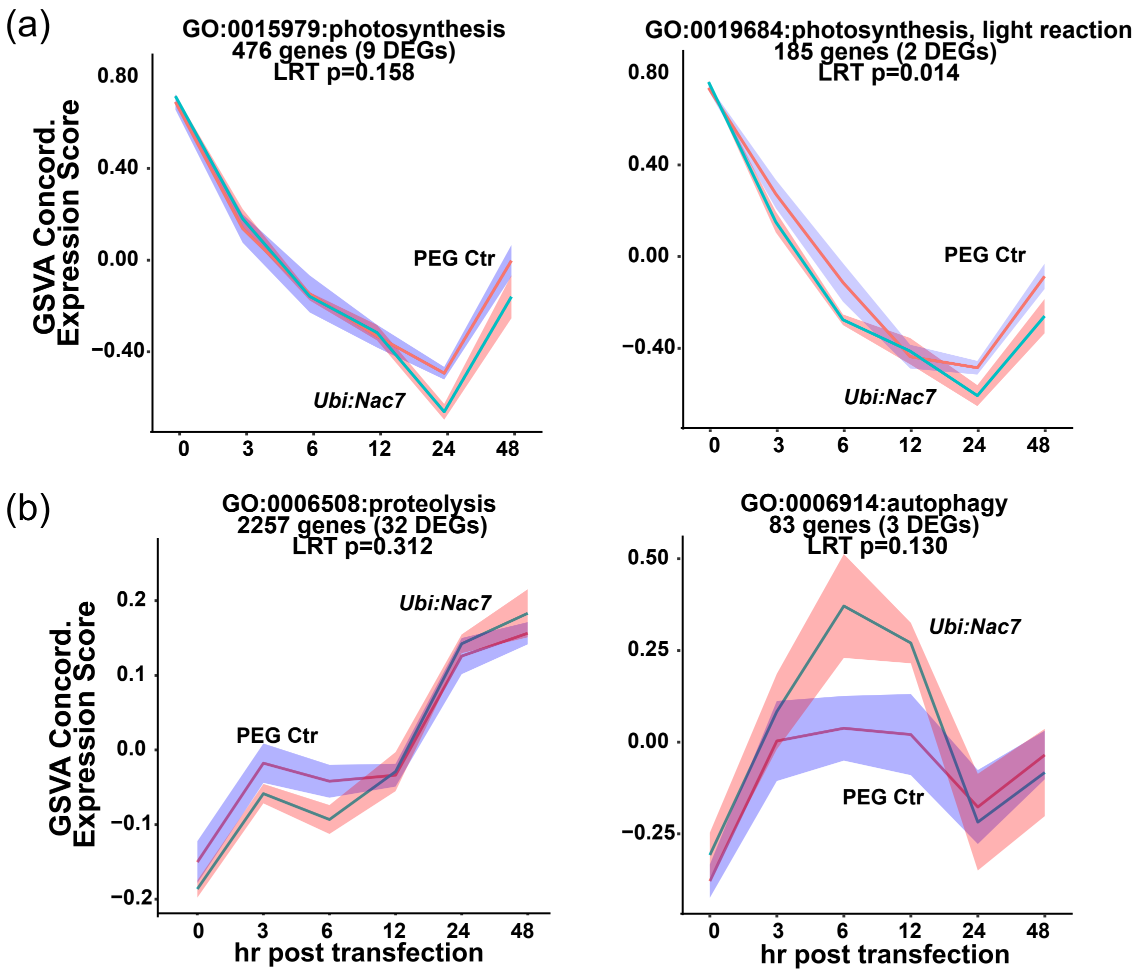

Supporting Figure 7 Gene Set Variation Analysis (GSVA) showed regulation of photosynthesis, autophagy and proteolysis pathways by NAC7 in protoplasts. GSVA determined concordant pathway expression profiles in protoplasts overexpressing *Nac7*. (a) Genes in the category for photosynthesis and light reaction pathways were negatively correlated with *Nac7* overexpression. (b) Genes in autophagy pathway were upregulated by *Nac7* overexpression, specifically during 3-12 hr post transfection. Proteolysis didn't show a clear upregulation with overexpression of *Nac7*. Red indicates protoplast overexpressing *Nac7* and blue indicates controls (n=4). 95% confidence intervals are shaded, with adjusted *p*-value for the LRT shown on the top.
