# Supplementary material for: Identification and characterization of a novel stay‐green QTL that increases yield in maize
Source: Plant Biotechnol J. 2019 May 20;17(12):2272–85. doi: 10.1111/pbi.13139 (PMC6835130; doi:10.1111/pbi.13139)
Supplement: Supplementary file 2 — Table S1 Sequence data from this article can be accessed under the following accession numbers. Table S2 List of DEGs in the nac7 node identified from nac7 RNAi leaves by RNAseq. [file PBI-17-2272-s002.docx]

**Supplementary Tables**

**Supporting Table 1.** Sequence data from this article can be accessed under the following accession numbers.

| **Species** | **Name** | **Accession No.** |
| --- | --- | --- |
| *Zea mays* | ZmNAC7 | Zm00001d041472  GRMZM2G114850 |
|  | ZmNAC1 | Zm00001d024268  GRMZM2G167018 |
|  | ZmNAP1 | Zm00001d028999  GRMZM2G011598 |
| *Arabidopsis thaliana* | NAC1 | At1g56010 |
|  | NAP | At1g69490 |
|  | ANAC019 | At1g52890 |
|  | ANAC016 | At1g34180 |
|  | ANAC072 | At4g27410 |
|  | ATAF1 | At1g01720 |
|  | AtJUB1 | At2g43000 |
|  | CUC1 | At3g15170 |
|  | NAM | At1g52880 |
|  | ORE1 | At5g39610 |
|  | ORS1 | At3g29035 |
|  | TIP | At5g24590 |
|  | VND1 | At2g18060 |
| *Oryza sativa* | OsSNAC1 | Os03g0815100 |
|  | OsNAC60 | Os12g0610600 |
|  | OsNAC104 | Os08g0200600 |
|  | OsNAC106 | Os08g0433500 |
|  | OsNAC15 | Os07g0684800 |
|  | OsNAC5 | Os11g0184900 |
|  | OsNAC6 | Os01g0884300 |
|  | OsNAP | Os03g0327800 |
| *Solanum lycopersicum* | SlORE1S02 | Solyc02g088180 |
|  | SlNAP2 | Solyc04g005610 |
| *Triticum* | TtNAM-B1 | DQ869673.1 |
|  | TaNAC-S | HM037184.1 |

**Supporting Table 2.** List of DEGs in the *nac7* node identified from *nac7* RNAi leaves by RNAseq. The adjusted *p*-value shows the Likelihood Ratio Test comparing gene expression between null and two *nac7* RNAi events.

| **B73v4 gene model** | **Annotation** | **Node** | **Padj** |
| --- | --- | --- | --- |
| Zm00001d041472_T001 | NAC7 | 85 | 2E-104 |
| Zm00001d041880_T002 | Beta-galactosidase 15-like protein | 85 | 9.3E-26 |
| Zm00001d018072_T001 | Membrane-anchored ubiquitin-fold protein, HCG-1 | 85 | 6.3E-22 |
| Zm00001d042451_T005 | Vacuolar protein sorting-associated protein 53 homolog-like protein | 85 | 1.5E-10 |
| Zm00001d006270_T009 | Enoyl-CoA hydratase 2, peroxisomal-like protein | 85 | 1.6E-09 |
| Zm00001d002319_T001 | Lecithine-cholesterol acyltransferase-like 4 | 85 | 3.1E-09 |
| Zm00001d044628_T001 | NAD(P)H oxidase | 85 | 1.5E-08 |
| Zm00001d028909_T001 | Peroxisomal enoyl-CoA hydratase/isomerase family protein | 85 | 1.8E-06 |
| Zm00001d030424_T003 | Auxin-induced protein 5NG4-like protein | 85 | 3.7E-06 |
| Zm00001d021506_T001 | Alanine synthase1 unknown beta-ureidopropionase | 85 | 3.3E-05 |
| Zm00001d023371_T001 | Saposin-like type B, region 2 | 85 | 0.00033 |
| Zm00001d042437_T001 | Copine III-like protein | 85 | 0.00041 |
| Zm00001d017704_T001 | VHS subgroup protein | 85 | 0.00043 |
| Zm00001d045372_T002 | Iron-sulfur assembly protein IscA, chloroplastic-like protein | 85 | 0.00062 |
| Zm00001d016949_T001 | Endoplasmic reticulum membrane protein | 85 | 0.00219 |
| Zm00001d004301_T001 | Inorganic phosphate transporter 1-4-like protein | 85 | 0.00244 |
| Zm00001d011525_T002 | Mitogen-activated protein kinase 10-like protein | 85 | 0.0043 |
| Zm00001d052734_T004 | Peptidase S54, rhomboid domain protein | 85 | 0.00454 |
| Zm00001d052211_T001 | UDP-glycosyltransferase 85A2-like protein | 85 | 0.00577 |
| Zm00001d021231_T002 | Inactive rhomboid protein 1-like protein | 85 | 0.00614 |
| Zm00001d036001_T008 | Peroxisomal biogenesis factor 11 (PEX11) | 85 | 0.01495 |
| Zm00001d005372_T015 | Protein serine/threonine phosphatase | 85 | 0.01531 |
| Zm00001d044951_T001 | Auxin-induced protein 5NG4-like protein | 85 | 0.01747 |
| Zm00001d024908_T003 | Arginase | 85 | 0.02306 |
| Zm00001d034935_T014 | Glutathione S-transferase/chloride channel, C-terminal protein | 85 | 0.02673 |
| Zm00001d038954_T004 | MRNA (guanine-N(7)-)-methyltransferase | 85 | 0.0278 |
| Zm00001d032815_T011 | Glutamine synthetase/guanido kinase, catalytic domain protein | 85 | 0.02792 |
| Zm00001d018049_T008 | HVA22-like protein a-like protein | 85 | 0.03477 |
| Zm00001d003664_T001 | Function unknown | 85 | 0.04242 |
